# A perspective on the mechanisms of herbal medicine for cognitive impairment

**DOI:** 10.3389/fneur.2025.1610542

**Published:** 2025-07-04

**Authors:** Si-yuan Zheng, Xiao-qing Zhou

**Affiliations:** ^1^Department of Encephalopathy, First Affiliated Hospital of Henan University of Chinese Medicine, Zhengzhou, China; ^2^Encephalopathy Center, First Clinical Medical School of Henan University of Chinese Medicine, Zhengzhou, China; ^3^Department of Acupuncture and Moxibustion, Beijing University of Chinese Medicine Shenzhen Hospital (Longgang), Shenzhen, China

**Keywords:** cognitive impairment, dementia, herbal medicine, mechanism, perspective

## Abstract

Cognitive impairment (CI) represents a critical public health burden exacerbated by aging populations and inadequate therapeutic options. Conventional treatments usually target single molecules, which limits their effectiveness in addressing the complex pathology of CI. In contrast, herbal medicine provides a systems-level therapeutic approach by simultaneously regulating multiple signaling pathways. This narrative perspective summarizes recent evidence on the pharmacological mechanisms through which herbal therapies mitigate CI. A focused literature review was performed to identify preclinical and clinical studies that emphasize the regulation of key pathways, including PI3K/Akt, Nrf2/HO-1, NF-κB, and BDNF/TrkB. These pathways act synergistically to reduce oxidative damage, inhibit pro-inflammatory cytokine production, and promote neuroplasticity. Representative compounds such as ginsenosides, catalpol, and standardized extracts from *Ginkgo biloba* and *Huperzia serrata* exhibit promising effects on these molecular pathways. Compared with monotherapies, herbal medicines offer a broader pharmacodynamic spectrum and potentially fewer adverse effects. These findings support the integration of herbal medicine into treatment strategies for CI and emphasize the need for high-quality clinical trials and mechanistic studies to validate and optimize its application.

## 1 Introduction

### 1.1 Epidemiology and socioeconomic burden of cognitive impairment

Cognitive impairment (CI), characterized by memory decline, executive dysfunction, and impaired daily living abilities, encompasses a spectrum of neurodegenerative disorders, including mild cognitive impairment and dementia (1). With the accelerating aging of the global population, the prevalence of CI has risen dramatically. According to the World Health Organization, approximately 55 million people worldwide currently live with dementia, a figure projected to reach 139 million by 2050 (2). China, experiencing one of the fastest aging rates, accounts for over 25% of global cases, with more than 15 million affected individuals, imposing a severe burden on public health and socioeconomic systems (3). CI not only drastically diminishes patients’ quality of life but also incurs exorbitant healthcare costs (1). In 2019, global dementia-related expenditures reached 1.3 trillion and are expected to surge to1.3 trillion and are expected to surge to 2.8 trillion by 2030 (4). Furthermore, the long-term caregiving burden on families and associated psychosocial issues underscore the urgency of addressing this condition.

### 1.2 Limitations of current managements

Current treatments for CI primarily focus on symptom management, with key pharmacological interventions including cholinesterase inhibitors (e.g., donepezil, rivastigmine) and N-methyl-D-aspartate receptor antagonists (e.g., memantine) (1). Although these drugs may temporarily alleviate certain symptoms, their efficacy is limited: cholinesterase inhibitors are effective in only 30–50% of patients, with benefits lasting an average of 6–12 months, while memantine, though modulating glutamatergic neurotransmission, offers marginal cognitive improvement in moderate-to-severe cases (5, 6). Additionally, these drugs often induce adverse effects such as gastrointestinal disturbances (e.g., nausea, diarrhea), cardiovascular complications (e.g., bradycardia), and neuropsychiatric symptoms (e.g., hallucinations), leading to poor patient adherence (5, 6). Crucially, existing therapies only slow disease progression without reversing neurodegeneration (5). Recent advances in monoclonal antibodies targeting *β*-amyloid (e.g., aducanumab) have shown promise, but their clinical benefits remain controversial, and they carry significant risks, such as cerebral edema (7). Thus, there is an urgent need to explore safer and more effective alternatives.

### 1.3 Growing interest and unique advantages of herbal medicine in CI management

Given these limitations, herbal medicine has gained increasing attention due to its holistic approach—characterized by multi-component, multi-target, and systemic regulatory mechanisms (8–10). Herbal medicine formulations (e.g., Danggui-Shaoyao-San, Kaixin-San) and bioactive compounds (e.g., *ginkgo biloba* extract, ginsenosides) have demonstrated neuroprotective, anti-inflammatory, antioxidant, and synaptogenic effects in preclinical and clinical studies (11–13). For instance, Huanglian-Jiedu-Tang may mitigate neuroinflammation by suppressing the Nuclear Factor kappa-B (NF-κB) pathway, while gastrodin enhances synaptic plasticity via BDNF/Tropomyosin receptor kinase B (TrkB) signaling (14, 15). Compared to western drugs, herbal medicine offers distinct advantages (16–18): (1) synergistic multi-target effects that address both symptoms and pathogenesis; (2) fewer side effects, supporting long-term use; and (3) personalized treatment strategies aligned with the heterogeneous nature of CI. Advances in network pharmacology and metabolomics have further elucidated the scientific basis of herbal medicine formulations, facilitating their global acceptance.

### 1.4 Objectives and significance

This study aims to systematically evaluate the pharmacological mechanisms and clinical evidence supporting herbal medicine in CI treatment, emphasizing its comparative advantages over Western medicine. It also addresses challenges in herbal medicine development, such as compositional complexity and quality control standardization. The scientific significance lies in providing a theoretical foundation for integrated Western and herbal medicine approaches, while the societal impact involves promoting cost-effective, low-toxicity natural therapeutics to alleviate the healthcare burden of aging populations. By integrating evidence-based medicine with herbal medicine’s holistic principles, this research may pave the way for novel CI intervention strategies.

## 2 Pharmacologically active components of herbal medicine in cognitive impairment management

Herbal medicine offers a multi-target therapeutic approach for cognitive impairment through various bioactive compounds derived from single herbs and complex formulations (8–10) (Table 1). Ginseng-derived ginsenosides (Rg1, Rb1) demonstrate neuroprotective effects by modulating the Nrf2/Heme oxygenase-1 (HO-1) antioxidant pathway and suppressing NF-κB-mediated neuroinflammation, while simultaneously promoting neurogenesis via PI3K/Akt signaling activation (19, 20). *Ginkgo biloba* flavonoids, particularly EGb761 extract, exhibit dual mechanisms of enhancing cerebral microcirculation and inhibiting amyloidogenic processes through ginkgolides regulate glycogen synthase kinase-3 beta (GSK-3β) regulation and tau phosphorylation modulation (21). The natural cholinesterase inhibitor huperzine A presents comparable efficacy to synthetic counterparts with improved gastrointestinal tolerability, offering a promising alternative for cholinergic enhancement.

**Table 1:** Bioactive compounds in herbal medicine and their mechanisms of action.

Compound name	Source herb	Mechanisms of action	Associated diseases
Ginsenoside Rg1	*Panax ginseng*	Inhibits NLRP3 inflammasome; activates PI3K/Akt and Nrf2/HO-1 pathways; enhances neurogenesis and synaptic plasticity.	AD, CI
Ginsenoside Rb1	*Panax ginseng*	Enhances dendritic spine density via miR-134; modulates BDNF/TrkB signaling.	AD, PD
Huperzine A	*Huperzia serrata*	Natural acetylcholinesterase inhibitor; increases synaptic acetylcholine levels.	AD, VD
Morroniside	*Cornus officinalis*	Inhibits TLR4/NF-κB signaling; upregulates BDNF/CREB pathway.	AD, CI
Verbenalin	*Verbena officinalis*	Downregulates BACE1; attenuates NF-κB-mediated neuroinflammation.	AD
Cornuside	*Cornus officinalis*	Promotes mitophagy; inhibits NLRP3 inflammasome via RAGE/TXNIP/NF-κB axis.	AD, CI
Catalpol	*Rehmannia glutinosa*	Activates BDNF–TrkB pathway; inhibits NF-κB-driven inflammation.	CI
Rehmannioside A	*Rehmannia glutinosa*	Reduces oxidative stress and ferroptosis via PI3K/Akt/Nrf2 and SLC7A11/GPX4 pathways.	VD
Geniposidic Acid	*Eucommia ulmoides*	Activates PI3K/Akt/GAP43 pathway; promotes neuronal regeneration.	AD
Aucubin	*Plantago asiatica*	Inhibits ERK-FOS inflammation; enhances autophagy via AMPK/mTOR pathway.	AD, ischemic stroke
Baicalin	*Scutellaria baicalensis*	Suppresses NF-κB pathway; reduces pro-inflammatory cytokines.	AD, neuroinflammation
Icariin	*Epimedium* spp.	Upregulates hippocampal BDNF; activates TrkB/CREB pathway and synaptic proteins (synaptophysin).	AD, synaptic dysfunction
EGb761 (Ginkgo extract)	*Ginkgo biloba*	Regulates GSK-3β to inhibit tau hyperphosphorylation; enhances cerebral microcirculation.	AD, VD
Ligustrazine	*Ligusticum chuanxiong*	Improves vascular endothelial function; increases cerebral blood flow via ACE inhibition.	VD, stroke
Puerarin	*Pueraria lobata*	Upregulates GLUT1/3 to enhance glucose transport; optimizes neuronal energy supply.	AD, metabolic impairment

AD, Alzheimer’s disease; CI, cognitive impairment; PD, Parkinson’s disease; VD, vascular dementia; BDNF, brain-derived neurotrophic factor; NF-κB, nuclear factor kappa-B; NLRP3, NOD-like receptor family, pyrin domain containing 3; PI3K, Phosphoinositide 3-kinase; Akt, Ak strain transforming; Nrf2, Nuclear factor erythroid 2-related factor 2; HO-1, heme oxygenase-1; TLR4, toll-like receptor 4; TrkB, tropomyosin receptor kinase B; CREB, cAMP response element-binding protein; IL, interleukin; SLC7A11, Solute Carrier Family 7 Member 11; GPX4, Glutathione Peroxidase 4; GAP43, growth associated protein 43; ERK-FOS, Extracellular Signal-Regulated Kinase-FBJ Murine Osteosarcoma Viral Oncogene Homolog; AMPK/mTOR, AMP-activated protein kinase/Mechanistic Target of Rapamycin; GSK-3β, Glycogen Synthase Kinase-3 beta; GLUT1, Glucose Transporter 1.

The synergistic potential of herbal medicine formulations is exemplified by Liuwei Dihuang Wan’s regulation of the kidney-brain axis through morroniside-mediated brain-derived neurotrophic factor (BDNF) upregulation and cAMP Response Element-Binding Protein (CREB) pathway activation, demonstrating significant neuroprotective effects against Aβ-induced toxicity (22). Kaixin San’s multi-component system targets both pathological hallmarks (Aβ aggregation) and functional restoration (synaptic plasticity) via BDNF/TrkB signaling while concurrently modulating the gut-brain axis, illustrating herbal medicine’s holistic therapeutic strategy (23, 24). Similarly, BuShen-YiQi (BSYQ), formulated to tonify Kidney essence and strengthen *Qi*, has shown therapeutic potential in alleviating cognitive impairment and delaying neurodegeneration. Preclinical studies indicate that BSYQ confers neuroprotective effects in cerebral ischemia–reperfusion models by activating the PI3K/Akt pathway, thereby enhancing neuronal survival and reducing infarct volume (25). In Alzheimer’s disease (AD) models, BSYQ mitigates Aβ-induced neurotoxicity, preserves blood–brain barrier integrity, and facilitates Aβ clearance by regulating transport proteins such as P-glycoprotein, low-density lipoprotein receptor-related protein 1, and the receptor for advanced glycation end products (RAGE) (26). Transcriptomic analysis further suggests that BSYQ modulates microRNAs involved in oxidative stress responses, synaptic signaling, and neuronal function, providing a molecular basis for its neuroprotective and anti-aging effects (27). Clinically, randomized controlled trials have demonstrated that BSYQ alleviates oxaliplatin-induced peripheral neuropathy, highlighting its neuroprotective potential in both central and peripheral nervous system disorders (28).

Iridoid compounds—including morroniside, verbenalin, cornuside, catalpol, rehmannioside A, geniposidic acid, and aucubin—exert neuroprotective effects by modulating oxidative stress, suppressing neuroinflammation, and enhancing synaptic plasticity. Morroniside, a bioactive component of *Cornus officinalis*, alleviates sevoflurane-induced cognitive dysfunction in aged mice through inhibition of the TLR4/NF-κB signaling pathway (29). Verbenalin, extracted from *Verbena officinalis*, decreases amyloid-beta accumulation by downregulating BACE1 and attenuating NF-κB-mediated inflammation in AD models (30, 31). Cornuside, another constituent of *Cornus officinalis*, enhances cognitive performance by promoting mitophagy, inhibiting NLRP3 inflammasome activation, and suppressing oxidative and inflammatory responses via the RAGE/TXNIP/NF-κB signaling axis (32–34). Catalpol reduces LPS-and isoflurane-induced cognitive impairments by inhibiting NF-κB-driven inflammation and facilitating synaptic recovery through activation of the BDNF–TrkB pathway (35–37). Rehmannioside A, derived from Rehmannia glutinosa, alleviates cognitive deficits in vascular dementia by reducing oxidative stress and inhibiting ferroptosis via the PI3K/Akt/Nrf2 and SLC7A11/GPX4 signaling pathways (38, 39). Geniposidic acid, found in *Eucommia ulmoides*, promotes neuronal regeneration and synaptic remodeling in AD models by activating the PI3K/Akt/GAP43 pathway (40). Aucubin, a compound from *Plantago asiatica* and *Aucuba japonica*, confers neuroprotection in ischemic and AD models by inhibiting ERK-FOS-mediated inflammation and promoting autophagic clearance through the AMPK/mTOR pathway (41, 42).

Current research challenges include the need for advanced standardization techniques to ensure batch-to-batch consistency in complex herbal mixtures and the requirement for more sophisticated model systems to validate multi-target mechanisms (43). Future investigations employing systems biology approaches could provide deeper insights into herbal medicine’s network pharmacology, potentially bridging the gap between traditional medicine and modern neurotherapeutics. The integration of rigorous quality control measures with cutting-edge neurobiological research methodologies may position herbal medicine as a valuable contributor to global cognitive disorder management strategies.

Unlike conventional Western drugs, which primarily target single molecular pathways, herbal medicine exerts effects through a systems-level mechanism that more effectively addresses the multifactorial etiology of cognitive impairment. Its primary pharmacological advantage is the simultaneous modulation of multiple signaling pathways—such as PI3K/Akt, Nrf2/HO-1, NF-κB, and BDNF/TrkB—that together regulate oxidative stress, neuroinflammation, and synaptic plasticity (19, 20, 23, 24). For instance, ginsenosides and catalpol activate the PI3K/Akt pathway to enhance neuronal survival by inhibiting apoptosis, while the Nrf2/HO-1 pathway increases antioxidant capacity and reduces oxidative damage caused by reactive oxygen species (19, 20). These pathways interact with neurotrophic systems, particularly the BDNF/TrkB axis, to promote synaptic repair and restore cognitive function at both cellular and network levels (23, 24). By targeting multiple pathways simultaneously, herbal formulations provide broader therapeutic effects and may reduce the risk of drug resistance and adverse effects commonly associated with Western monotherapies. Therefore, the integrative modulation of interconnected signaling pathways highlights the superior pharmacodynamic profile of herbal medicine in managing complex neurodegenerative disorders such as cognitive impairment.

Based on the synthesized evidence, we propose the concept of “integrative neuro-homeostasis” to describe the therapeutic rationale underlying the use of herbal medicine in cognitive disorders. Instead of solely focusing on multitarget engagement, we suggest that herbal formulations achieve their therapeutic effects through the coordinated modulation of four interrelated pathological domains: oxidative stress, neuroinflammation, synaptic dysfunction, and cerebral energy metabolism. This conceptual framework is supported by a comparative analysis of existing preclinical and clinical studies, which consistently indicate that interventions targeting a single domain yield limited therapeutic efficacy, whereas those modulating multiple domains result in more sustained improvements in cognitive function. By articulating this network-based hypothesis, we move beyond descriptive summaries and propose a clear conceptual framework to guide future mechanistic and translational research.

## 3 Mechanisms of herbal medicine in treating cognitive impairment

### 3.1 Anti-neuroinflammatory and antioxidant effects

**Figure 1:**
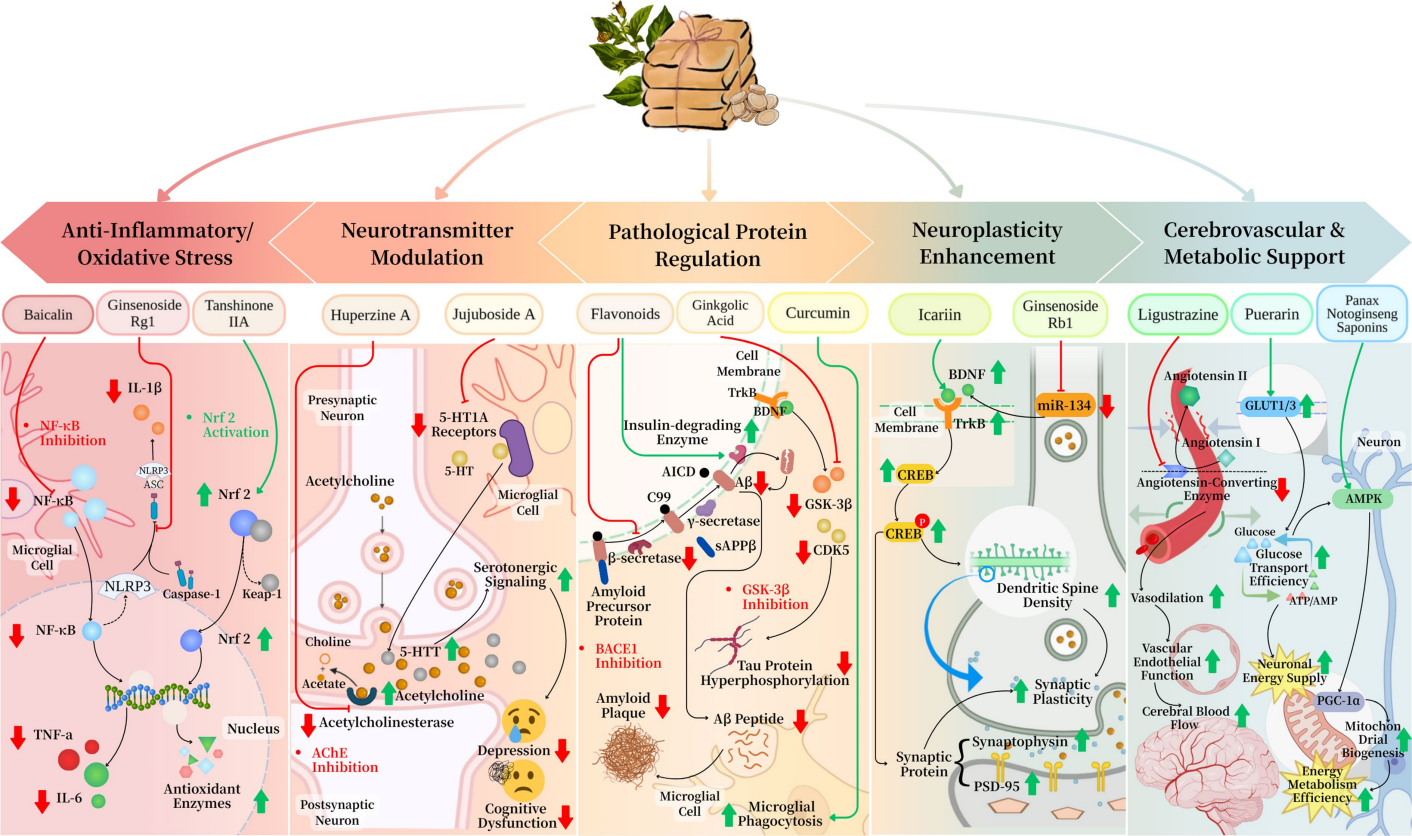
Multi-target mechanisms of herbal medicine in cognitive impairment.

Herbal medicine exerts neuroprotective effects through multi-target regulation of neuroinflammation and oxidative stress (Figure 1). Current research demonstrates that baicalin from Huanglian Jiedu Decoction significantly inhibits NF-κB signaling pathway activation, reducing the release of pro-inflammatory cytokines including tumor necrosis factor-*α* and interleukin (IL)-6 (44, 45). Simultaneously, ginsenoside Rg1 modulates the assembly and activation of NLRP3 inflammasomes, decreasing caspase-1-mediated IL-1β maturation and secretion (46). Regarding antioxidant effects, tanshinone IIA activates the Nrf2/HO-1 pathway, enhancing the activity of superoxide dismutase and glutathione peroxidase to effectively scavenge oxygen free radicals (47). This dual regulatory mechanism provides a stable microenvironment for neuronal cells and delays neurodegenerative progression.

### 3.2 Modulation of neurotransmitter systems

Herbal medicine exhibits regulatory effects on multiple neurotransmitter systems (Figure 1). Huperzine A, as a natural acetylcholinesterase inhibitor, significantly increases synaptic acetylcholine concentration and improves cholinergic neurotransmission deficits (48). Furthermore, jujuboside A modulates 5-HT1A receptor activity to alleviate cognitive dysfunction associated with depression (49). This synergistic regulation of multiple neurotransmitter systems demonstrates more comprehensive therapeutic effects compared to single-target Western medications.

### 3.3 Inhibition of pathological protein aggregation

In AD pathology regulation, flavonoids inhibit *β*-secretase (β-site amyloid precursor protein cleaving enzyme, BACE1) activity to reduce Aβ generation while promoting insulin-degrading enzyme expression to accelerate Aβ clearance (50). GSK-3β and CDK5 activity to decrease tau protein hyperphosphorylation (51). Notably, certain herbal medicine components like curcumin enhance microglial phagocytosis of Aβ, achieving bidirectional regulation of pathological proteins (Figure 1).

### 3.4 Promotion of neuroregeneration and synaptic plasticity

Herbal medicine facilitates neural repair through neurotrophic factor signaling networks (Figure 1). Icariin significantly upregulates hippocampal BDNF expression and activates the TrkB/CREB signaling pathway, increasing synaptic protein expression including synaptophysin and PSD-95 (52, 53). Ginsenoside Rb1 enhances dendritic spine density through miR-134-mediated mechanisms to improve synaptic plasticity (54). These effects extend beyond symptomatic relief to promote neural network reconstruction and functional compensation.

### 3.5 Improvement of cerebral blood flow and energy metabolism

Herbal medicine demonstrates unique advantages in regulating cerebrovascular function and energy metabolism (Figure 1). Ligustrazine inhibits angiotensin-converting enzyme to improve vascular endothelial function and increase cerebral blood flow (55). Puerarin upregulates GLUT1/3 expression to enhance glucose transport efficiency and optimize neuronal energy supply (56, 57). Panax notoginseng saponins regulate the AMPK/PGC-1α pathway to promote mitochondrial biogenesis and improve energy metabolism efficiency (58). This multi-level regulation provides essential material foundations for cognitive function recovery.

In summary, through these multi-target, multi-pathway synergistic effects, herbal medicine constitutes a systematic therapeutic network for cognitive impairment. However, current research still faces challenges including complex compositions and narrow effective concentration ranges. Future studies should incorporate novel technologies like organoid culture and single-cell sequencing to elucidate herbal medicine’s comprehensive regulatory mechanisms, providing theoretical foundations for developing new neuroprotective agents. Meanwhile, establishing biomarker-based efficacy evaluation systems will facilitate the internationalization of herbal medicine in cognitive disorder treatment.

## 4 Application of modern technologies in the study of herbal medicine mechanisms

### 4.1 Network pharmacology and target prediction

Network pharmacology has transformed the study of traditional herbal medicine by enabling systematic mapping of complex herb-compound-target-disease interactions (59). By integrating cheminformatics and bioinformatics, computational algorithms construct multi-scale networks that reveal the polypharmacological nature of herbal medicine formulations (60). For example, network analysis of *Ginkgo biloba* has identified a variety of bioactive compounds interacting with cognitive impairment-related targets, including *APP*, *AKT1*, and *PTGS2*, highlighting its multi-target mechanisms (61). Machine learning techniques, such as deep neural networks, further enhance predictive accuracy by analyzing structure–activity relationships across extensive phytochemical libraries (62). These approaches not only validate traditional uses but also uncover novel therapeutic targets for cognitive disorders.

### 4.2 Multi-omics integration (transcriptomics, proteomics, metabolomics)

Multi-omics technologies provide a comprehensive framework for deciphering herbal medicine’s holistic effects on cognitive function. Transcriptomic profiling of hippocampal tissue after herbal medicine treatment reveals differential expression of neuroplasticity-related genes (e.g., BDNF), while high-resolution mass spectrometry-based proteomics detects modulation of synaptic proteins (e.g., synaptophysin) and pathological markers (e.g., phosphorylated tau) (63). Metabolomic analyses, particularly of cerebrospinal fluid, capture dynamic changes in neurotransmitters (e.g., acetylcholine, glutamate) and energy metabolites (e.g., lactate), offering functional insights into herbal medicine efficacy (64). Integrated multi-omics studies have identified critical regulatory nodes, such as the CREB-miR-132-BDNF axis, which mediates herbal medicine-induced synaptic enhancement and neuroprotection.

### 4.3 Molecular docking and structural biology

Computational molecular docking, combined with advanced structural biology techniques, elucidates atomic-level interactions between herbal medicine compounds and their molecular targets (65). For instance, ginsenoside Rg1 has been shown to stabilize TrkB receptor activation by binding to the BDNF dimerization interface (66). Virtual screening of herbal medicine libraries against BACE1 identified salvianolic acid B as a potent inhibitor, with X-ray crystallography revealing its unique binding mode distinct from synthetic drugs (67). These structural insights facilitate the rational optimization of herbal medicine-derived lead compounds while preserving their inherent multi-target properties, bridging traditional knowledge with modern drug discovery.

### 4.4 Advanced imaging technologies

Multimodal imaging enables real-time, non-invasive assessment of herbal medicine effects on brain structure and function (68). Ultra-high-field 7 T magnetic Resonance Imaging quantifies hippocampal volume preservation and white matter integrity in AD models treated with herbal medicine (69). Two-photon microscopy, using amyloid-specific probes, dynamically visualizes plaque clearance rates, while novel Positron Emission Tomography tracers track tau pathology modulation (70). Optoacoustic imaging further reveals rapid cerebrovascular improvements, such as enhanced cortical perfusion within 30 min post-herbal medicine administration (71). These technologies provide spatially and temporally resolved evidence of herbal medicine’s neuroprotective and restorative effects.

### 4.5 Technological convergence and future directions

The synergy of network pharmacology, multi-omics, structural biology, and advanced imaging is forging a new paradigm in herbal medicine research. Network analyses, validated by multi-omics datasets, are decoding the systems-level mechanisms of complex herbal medicine formulations (72). High-content screening platforms now enable multiplexed assessment of cellular responses in herbal medicine-treated neuronal models (73). Emerging tools like spatial transcriptomics promise to map region-specific gene expression changes induced by herbal medicine interventions (74). However, challenges persist in standardizing herbal medicine preparations for reproducible omics studies and developing human-relevant organoid models for mechanistic validation. Addressing these gaps will accelerate the translation of herbal medicine research into clinically viable therapies for cognitive impairment.

## 5 Challenges and future perspectives

### 5.1 Current research limitations

Despite the demonstrated potential of herbal medicine in cognitive disorder treatment, significant challenges remain. First, the inherent complexity of herbal medicine compositions presents difficulties in active ingredient identification, quality control, and standardization (75). A single herbal extract may contain hundreds of compounds, while multi-herb formulations introduce additional complexities due to potential synergistic or antagonistic interactions. Second, mechanistic studies often lack depth, with most research limited to phenotypic observations or single-pathway validation rather than comprehensive analyses of multi-target regulatory networks (76). For instance, while flavonoids have been shown to inhibit Aβ aggregation, their integrated effects on synaptic plasticity, neuroinflammation, and blood–brain barrier function remain incompletely characterized. Furthermore, clinical studies frequently suffer from methodological limitations, including small sample sizes, non-standardized treatment protocols, and insufficient long-term follow-up, resulting in lower levels of evidence-based validation. These factors collectively hinder global recognition and clinical translation of herbal medicine research.

### 5.2 Future research directions

Future investigations should leverage cutting-edge technologies to address current limitations. At the basic research level, computational approaches such as virtual screening and target prediction can accelerate the discovery of bioactive compounds, while organ-on-chip platforms and single-cell sequencing may elucidate spatial heterogeneity in neuroprotective mechanisms. For example, network pharmacology approaches exploring the “herbal medicine-gut microbiota-brain axis” may reveal novel pathways through which herbal formulations modulate cognitive function. In clinical translation, precision medicine strategies should be prioritized, utilizing biomarker stratification to enable personalized treatment regimens. Additionally, advanced drug delivery systems (e.g., exosome-based carriers) may enhance blood–brain barrier penetration and target specificity. Standardization and internationalization efforts are equally critical, requiring establishment of quality control systems based on chromatographic fingerprinting and adherence to international consensus guidelines for multicenter randomized controlled trials.

### 5.3 Translational medicine potential

The integration of herbal medicine formulations with modern targeted therapies represents a promising paradigm for cognitive disorder treatment. On one hand, structural optimization of active ingredients (e.g., ginsenoside Rg3-EE derivatives) could yield compounds with both polypharmacological profiles and improved bioavailability. On the other hand, exploring combination therapies with monoclonal antibodies (e.g., aducanumab) or gene therapies (e.g., BACE1-targeting siRNA) may produce synergistic effects while mitigating drug resistance. For instance, combined use of huperzine A with donepezil has demonstrated prolonged cognitive enhancement in clinical observations. Critical infrastructure development includes humanized AD mouse models, cerebrospinal fluid biobanks, and real-world data analytics systems to facilitate bidirectional bench-to-bedside translation. Ultimately, the convergence of herbal medicine’s holistic regulation with Western medicine’s targeted interventions may pioneer next-generation therapeutic strategies for cognitive disorders.

In summary, herbal medicine research for cognitive disorders is transitioning from empirical practice to mechanism-driven science. Overcoming current limitations will require multidisciplinary innovation, with future advancements potentially contributing not only to herbal medicine modernization but also to global neurodegenerative disease therapeutics.

## 6 Summary

Herbal medicine offers a promising systems-level approach to prevent and treat CI by targeting multiple pathogenic mechanisms simultaneously. In contrast to conventional drugs that act on single targets, herbal compounds exert therapeutic effects by coordinately modulating multiple signaling pathways. This study highlights four essential signaling pathways—PI3K/Akt, Nrf2/HO-1, NF-κB, and BDNF/TrkB—as core mediators of the neuroprotective effects of herbal therapies. These pathways jointly contribute to antioxidative, anti-inflammatory, and neuroplasticity-promoting effects that help reduce neuronal damage and preserve cognitive function. Compounds such as ginsenosides, catalpol, and standardized extracts of *Ginkgo biloba* and *Huperzia serrata* have demonstrated regulatory activity on these pathways in preclinical and clinical studies. The integrated pharmacological effects of herbal medicine provide broader therapeutic potential and may reduce the risk of resistance and adverse effects often seen with monotherapies. Although narrative in format, this review incorporates updated literature to strengthen the mechanistic understanding of how herbal therapies improve cognitive function. Future studies should prioritize rigorous randomized controlled trials and mechanistic research to validate current findings and promote the clinical application of herbal medicine in neurodegenerative disease management.

## Data Availability

The original contributions presented in the study are included in the article/supplementary material, further inquiries can be directed to the corresponding author.
